# Effect of Stem Cells from Human Exfoliated Deciduous Teeth Spent Medium (SHED-SM) on the Healing of Traumatic Buccal Mucosal Ulcers

**DOI:** 10.12688/f1000research.164339.2

**Published:** 2025-10-21

**Authors:** Juni Handajani, Hendrik Setia Budi, Lisa R. Amir, Sri Angky Soekanto

**Affiliations:** 1 Department of Oral Biology, Faculty of Dentistry, Universitas Gadjah Mada, Yogyakarta, Indonesia; 2 Department of Oral Biology, Faculty of Dentistry, Airlangga University, Surabaya, Indonesia; 3 Department of Oral Biology, Faculty of Dentistry, Indonesia University, Jakarta, Indonesia

**Keywords:** nanoemulgel, spent medium, Stem Cells from Human Exfoliated Deciduous Teeth (SHED), traumatic ulcers, wound healing.

## Abstract

**Introduction:**

Traumatic ulcers are among the most prevalent injuries with a reported incidence of up to 95%. This study aims to evaluate Stem Cells from Human Exfoliated Deciduous Teeth Spent Medium (SHED-SM) nanoemulgel on the viability cells of NIH-3T3 and buccal mucosal ulcer wounds.

**Materials and methods:**

The viability cells test of SHED-SM was analyzed on NIH-3T3 fibroblast cells using MTT. Thirty male Wistar rats, 2-3 months, were used as experimental subjects. Traumatic ulcer models were created by excising the right buccal mucosa with a 3 mm punch biopsy. Subjects were divided into 2 groups @15 rats for the treatment (SHED-SM) and control. The gel was applied on the surface once a day for 4 days. Evaluation was conducted on days 1, 3, 5, 7, and 14. Hematoxylin Eosin (HE) and immunohistochemical were stained to evaluate a number of neutrophils and the expression of COX-2, VEGF, and Col-1.

**Results:**

The one-way ANOVA results showed significant differences in the viability test, the number of neutrophils, and the expression of COX-2, Col-1, and VEGF. The number of neutrophils and the expression of COX-2, Col-1, and VEGF were higher in the treatment group compared to the control.

**Conclusion:**

SHED-SM demonstrates potential to accelerate the healing of traumatic buccal mucosal ulcers.

## Introduction

Ulcers are a type of injury of the oral mucosa caused by various forms of trauma, including thermal, mechanical, chemical, and electrical trauma. ^1^ The 2018 Riskesdas report stated that ulcers represent a significant dental and oral health issue in Indonesia, with a prevalence rate of 95%. ^2^ Clinically, ulcers are characterized by the loss of epithelium, often present with yellowish fibrin exudate at the center, erythematous edges, and a round shape. Their size varies depending on the duration, intensity, and cause. ^3^ The natural healing process of ulcerative wounds typically takes 10-14 days if the causative factor is eliminated. ^4^ Wound healing is a complex and long process involving multiple phases: hemostasis, inflammation, proliferation, and maturation. ^5^ The hemostasis phase occurs immediately after the injury, and is characterized by vasoconstriction, and platelet activation that binds collagen to the extracellular matrix (ECM), and initiate the blood clotting process. ^6^ Platelets then release chemical mediators, including transforming factor beta (TGF-β), platelet-derived growth factor (PDGF), and epidermal growth factor (EGF), which stimulate the macrophage migration, neutrophil infiltration, and fibroblast proliferation. ^6^

Regenerative therapies utilizing stem cells are being developed to accelerate wound healing. Stem cell products offer potential solutions to address deficiencies in components critical to the wound-healing process. The secretion products of stem cells, collectively referred to as the secretome, have garnered particular attention in this context. Stem cell products are expected to overcome the lack of components that can cause delays in the wound-healing process. The secretion products of stem cells, known as the secretome ^7^ have drawn particular attention in this context. Stem cells can be derived from several parts of the body, including dental tissues, such as Dental Pulp Stem Cells (DPSCs), Stem Cells from Human Exfoliated Deciduous Teeth (SHED), Stem Cells from Apical Papilla (SCAP), Periodontal Ligament Stem Cells (PDLSCs), Dental Follicle Stem Cells (DFSCs), and Gingival Mesenchymal Stem Cells (GMSCs). ^8^

Stem cells originating from deciduous tooth pulp tissue are referred to as Stem Cells from Human Exfoliated Deciduous Teeth (SHED). SHED is known as a potential source of regenerative therapy due to its ability to differentiate into various cells, such as fibroblasts, odontoblasts, adipocytes, nerves, and epithelial cells. ^7^ Furthermore, SHED exhibits robust osteogenic and adipogenic differentiation capabilities and expresses several critical growth factors, including Fibroblast Growth Factor (FGF), Transforming Growth Factor (TGF), Connective Tissue Growth Factor (CTGF), Nerve Growth Factor (NGF), and Bone Morphogenetic Protein (BMP). ^9, 10^ The elevated expression of these growth factors facilitates tissue regeneration by promoting cell migration, proliferation, and differentiation. ^11^ SHED demonstrates a higher proliferation rate than other types of stem cells, even in unfavorable conditions, such as high glucose levels and hypoxic conditions. High expression of several growth factors can help accelerate tissue regeneration by stimulating the process of cell migration, proliferation, and differentiation. ^11^

Despite its potential, cell-based therapy still has shortcomings, such as the risk of cell mutations that lead to tumorigenesis and rejection during transplantation. ^12, 13^ To address these challenges, the use of stem cell secretion products, known as secretomes, is an alternative to overcome problems related to cell-based therapy. ^14^ Secretomes are molecules and biological factors secreted by cells into the extracellular environment that play pivotal roles in homeostasis, inflammation, angiogenesis, apoptosis, proteolysis, and signaling. ^15^ These products contain growth factors, cytokines, chemokines, extracellular matrix (ECM), and small molecules, including metabolites, microvesicles, and exosomes.

Secretomes can be obtained by isolating stem cells in growth media, resulting in a conditioned medium or spent medium. The use of spent medium as a culture medium offers several advantages since it is simple, practical, and affordable. ^16^ The process for obtaining spent medium is relatively safe and minimally invasive, which allows the collection of cell secretion without using and taking the cells themselves. ^17^ Based on the description above, developing a safe therapy method for buccal mucosal ulcer wounds using SHED-SM nanoemulgel is necessary.

SHED-SM is a conditioned medium of SHED enriched with growth factors, cytokines, exosomes, and other bioactive molecules that are at the core of secretome therapy. The nanoemulgel formulation functions as a topical vehicle that is thought to protect and regulate secretome release components, thereby enhancing stability, local retention, and therapeutic efficacy in oral or wound soft tissue applications. SHED-SM may accelerate wound healing by playing a role in the transition phase from hemostasis through controlling inflammation, shifting macrophage phenotype from proinflammatory M1 toward reparative M2, inducing angiogenesis and cell migration, and balancing collagen synthesis and provisional matrix formation.

## Material and methods

This research was conducted as part of the Indonesian Collaborative Research Program (Riset Kolaborasi Indonesia) according to Contract No. 1917/UN1/DITLIT/PT.01.03/2024 between Universitas Airlangga, Universitas Gadjah Mada, and Universitas Indonesia. This research has obtained ethical approval from the Ethics Commission of the Faculty of Dentistry and Prof. Soedomo Dental Hospital (RSGM Prof. Soedomo), Universitas Gadjah Mada University as documented in Letter No. 153/UN1/Kep/FKG-RSGM/EC/2024 dated July 25, 2024 for animal research.

The study method was laboratory experimental with separate in vitro and in vivo testing. In vitro cell viability testing was conducted at the Integrated Research Laboratory, Faculty of Dentistry, Gadjah Mada University, while in vivo testing was done at the Integrated Research and Testing Laboratory, Gadjah Mada University.

### Isolation of SHED and SHED-SM

This study was followed previous research and adhered to ethical guidelines, ensuring that written informed consent was obtained from donors. The isolation of SHED and SHED Spent Medium (SHED-SM) was carried out at Universitas Airlangga. The ethics approval was granted by the Committee for Medical and Health Research Ethics at the Faculty of Dental Medicine (0530/HRECC.FODM/V/2024) Universitas Airlangga dated May 17, 2024 for human participant. Deciduous teeth were extracted under sterile conditions in a surgical setting from two 6-year-old patients. The selection criteria for the tooth samples included anterior teeth without caries, pulp exhibiting a red coloration, absence of gum infection, and extraction performed in an aseptic environment. ^18^

Pulp tissues obtained from deciduous teeth were enzymatically digested using collagenase type I (4 mg/mL) and dispase (2 mg/mL) for one hour at 37°C before being plated onto a dish cell culture. The SHEDs were cultivated in alpha-minimum essential medium (α-MEM) Eagle, supplemented with 10% fetal bovine serum (FBS) and 1% penicillin/streptomycin. Cultures were cultivated in 35 mm dishes at 37°C in a humidified environment with 5% CO _2_. The growth media were replaced every two to three days until the cells reached confluence.

Microscopic observation confirmed that the SHEDs were cultivated to guarantee a uniform population with a spindle-like shape, characteristic of mesenchymal stem cells (MSCs). Subcultures were performed across several passages. Passage 2 cells were used for surface marker characterization and differentiation analysis, while passage 4 cells were used for SHED-SM isolation.

To prepare SHED-SM, SHED cells obtained from the Indonesian Collaborative Research project at the fourth passage were cultured in complete growth medium after being inoculated into a T-75 flask at a density of 2 million cells. After reaching 70% to 80% confluency, the cells were rinsed twice with phosphate-buffered saline (PBS), and the medium was replaced with serum-free alpha-MEM (BM). The cells were incubated for days 1, 3, and 5 at 37°C in a humidified atmosphere containing 5% CO _2_ to create SHED-SM (H1, H3, H5). This process generated 8–10 million SHEDs, equivalent to around 15 mL of SM. After collection, the SM was centrifuged at 1,200 rpm for 5 minutes, filtered through a 0.2-μm-pore-size filter and stored at -80°C. ^18^

### Viability cells-test of SHED-SM against NIH-3T3 fibroblast cells

Viability cells was measured using the MTT assay using a microplate reader at wavelength of 490 nm (Thermo Scientific). NIH-3T3 fibroblast cells suspended in RPMI 1640 medium (100 μl, density 1.5x10^4 cells/well) were put into a 96-well plate and incubated for 24 hours in a 5% CO _2_ incubator. Furthermore, SHED-SM was added to each well in three replications, followed by incubation at a 37°C in 5% CO _2_ incubator for 24 hours. After incubation, the medium in each well was discarded and replaced with a mixture of of SHED-SM and medium at a ratio of 50:150 μl, for a total of 200 μl. Finally, 10 μl of 0.5% MTT in the culture medium was added during the final stage of incubation. The plate was then incubated again at 37°C for 2.5 hours. Living cells formed purple formazan due to reaction with MTT. The formazan was dissolved in an SDS solution and incubated at room temperature for 12 hours.

### Treatment of experimental animals

Thirty Wistar rats aged 2-3 months, weighing 250-300 grams, were used as experimental subjects and adapted in individual cages for 3 days. The rats were then randomly divided into two groups of 15 each: the treatment group (SHED-SM nanoemulgel) and the control (Aloclair Plus) (Kalbe Farma). All experimental animals were treated at the Integrated Research and Testing Laboratory (LPPT) of Universitas Gadjah Mada.

The induction of traumatic ulcers began by anesthetizing the rats intramuscularly with ketamine at a dose of 0.1 ml/100 grams of body weight. A 3 mm diameter punch biopsy tool (Medax model Epitheasy) was then pressed and rotated on the buccal mucosa to create the ulcer. Therapy was performed once a day at 10:00 Western Indonesian Time for four consecutive days using a regular size 3.0 mm microbrush (Microbrush International) according to the treatment and control groups.

 Decapitation was performed on days 1, 3, 5, 7, and 14, with three rats sacrificed per time point after being anesthetized intramuscularly with ketamine HCl (Ketamil) at the same dose (0.1 mL/100 grams of body weight). Ulcer tissue was excised from the buccal mucosa with a thickness of ± 2-3 mm. The tissue samples were fixed in 10% Buffered Natural Formalin (BNF) solution for 24 hours.

Histology preparation was carried out at the Integrated Research Laboratory (LRT) of the Faculty of Dentistry, Universitas Gadjah Mada. The ulcer tissue specimens were dehydrated using an automatic tissue processor (Tissue-Tek II Sakura Timing Disc, USA) for 20 hours. The specimens were then cleared with xylene and infiltrated with paraffin. The embedding process was carried out in a tissue embedding cassette (Refurbished Sakura TECTM 4 Tissue Embedding Center), and tissue sections 3 μm thick were cut using a rotary microtome (Accu-Cut ^®^ SRMTM Rotary Microtome).

### Tissue staining

Tissue staining was performed using Hematoxylin Eosin (Harris Hematoxylin, Leica Biosystems, USA) and immunohistochemistry (Elabscience). Deparaffinization and rehydration procedures were conducted using graded xylol and alcohol solutions starting from 100%, 95%, 80% and 70% concentrations. The specimens were stained in hematoxylin solution for 5 minutes, then rinsing using running water for 3 minutes, and then stained with eosin solution for 1 minute, followed by another rinse with running water. Dehydration was performed with graded alcohol solutions at 70%, 80%, 96%, and 100% concentrations before being cleared using xylol and mounting.

Immunohistochemistry staining was performed using an IHC kit (Elabscience). Endogenous peroxidase activity was blocked by incubating the samples in 3% H _2_O _2_ at room temperature for 10 minutes, followed by washing with PBS. Normal Goat Blocking Buffer was applied to the specimens and incubated for 30 minutes at 37°C. The samples were then incubated overnight at 4°C with primary antibodies: anti-COX-2 (Rabbit Polyclonal antibody COX-2, NB100-689, Novusbio) 1:100, anti-Collagen 1 (Rabbit Polyclonal antibody Collagen 1, NB100-408, Novusbio) 1:100 and anti-VEGF (Rabbit Polyclonal antibody VEGF, sc-152, Santa Cruz Biotechnology) 1:50 as much as 50 μl of each antibody.

After washing with PBS, the samples were treated with Polyperoxidase-anti-Mouse/Rabbit IgG (E-IR-R217B) and incubated for 20 minutes at room temperature. Following another PBS wash, the samples were stained with the chromogen 3,3’-diaminobenzidine (DAB) in a 1:20 ratio with the substrate until a brown color appeared, indicating positive staining. The process concluded with dehydration using graded alcohol solutions, clearing with xylene, and mounting.

### Observation of neutrophil infiltration and immunohistochemistry

Neutrophil infiltration was observed by counting the number of neutrophil cells in the connective tissue of the wound edge using a light microscope at 400× magnification. Each slide was analyzed in three fields of view, and the results were averaged to determine the neutrophils count per field.

COX-2, VEGF, and Collagen 1 (Col-1) protein expression was observed in the connective tissue at the ulcer edge. COX-2 expression was identified by brown staining in the cytoplasm of neutrophils, macrophages, lymphocytes, fibroblasts, and the extracellular matrix. The number of COX-2-positive cells in the ulcer edge epidermis was counted across three fields of view.

Col-1 expression was indicated by brown staining in fibroblasts and the extracellular matrix, while VEGF expression was marked by brown staining in endothelial cells, fibroblasts, and inflammatory cells within the ulcer area. Measurements of VEGF and Col-1 expression were conducted using ImageJ software (National Institutes of Health, version 1.54) based on specimen photographs. The data obtained were analyzed using parametric statistical tests, including one-way ANOVA. The results of the data calculations showed homogeneity (Levene's test) and normality (Shapiro–Wilk test), then a post hoc LSD test was carried out, to determine significant differences.

## Results

**Figure 1.**
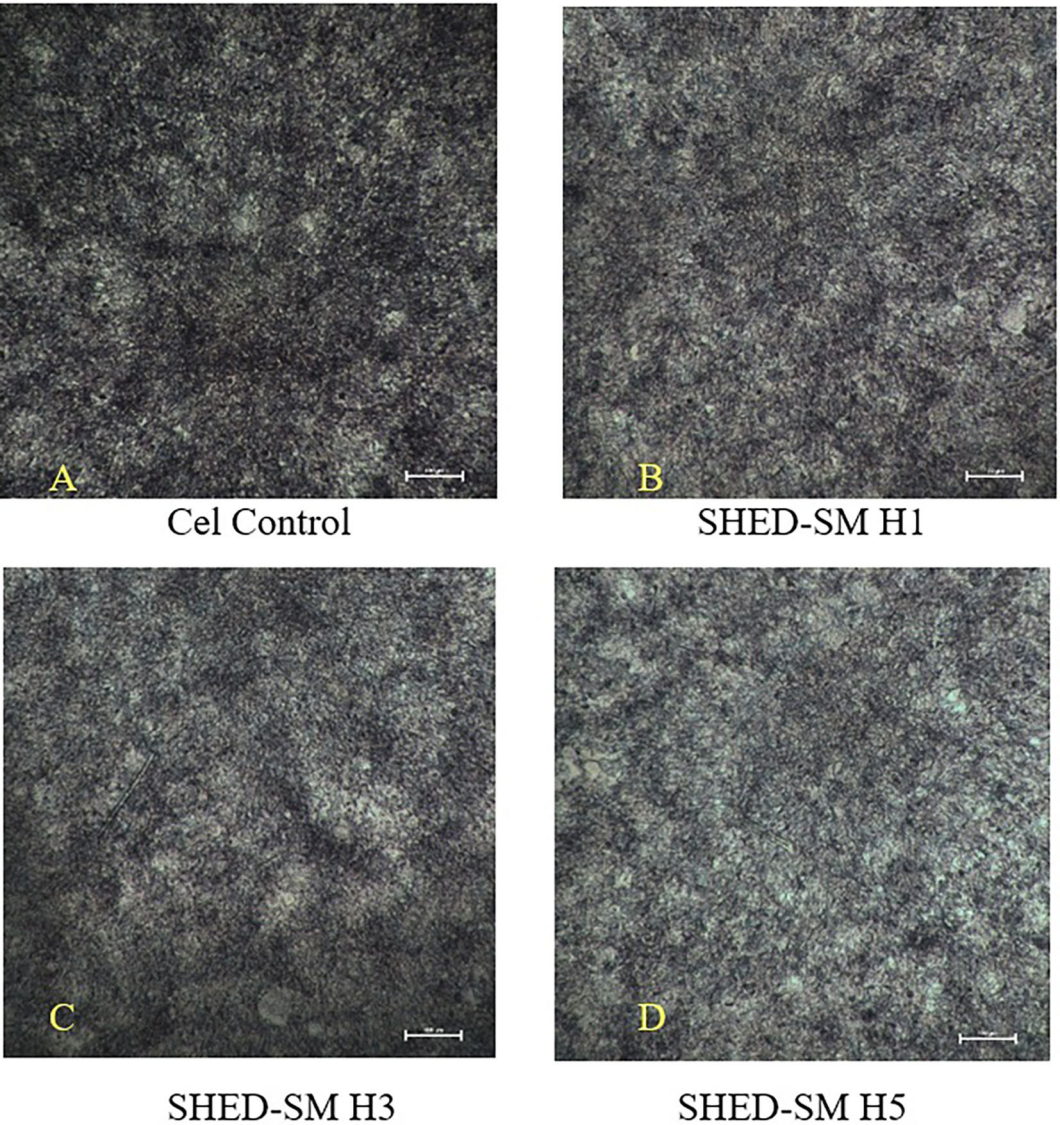
Image of NIH-3T3 fibroblast cells (A) and NIH-3T3 fibroblast cells after treatment using nanoemulgel spent medium SHED (SHED-SM) H1 (B), H3 (C) and H5 (D). Cell viability was indicated by black living cells and fuzzy crystals at the bottom of the well after MTT application. The highest viability of NIH-3T3 fibroblast cells was shown after applying SHED-SM H1.

The results of the viability cells test of SHED Spent Medium (SHED-SM) nanoemulgel against NIH-3T3 fibroblast cells after MTT administration are presented in Figure 1. After MTT application, black living cells and fuzzy crystals were visible at the bottom of the well.

**Figure 2.**
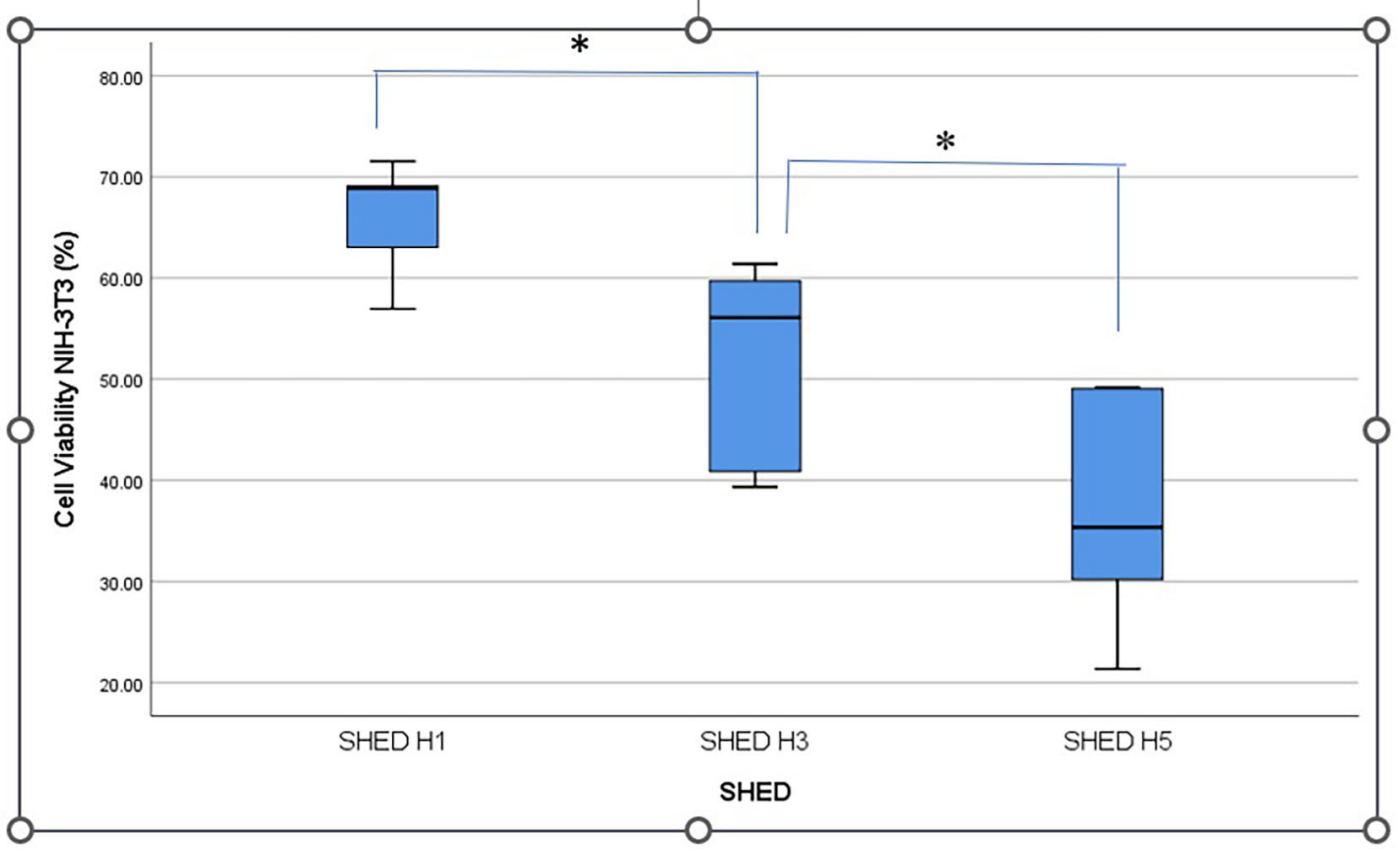
The highest percentage of NIH-3T3 fibroblast cell viability was shown after the application of nanoemulgel SHED-H1spent medium (SHED-SM H1) and significant differences in the comparison of each concentration (*) (p<0.05).

Figure 2 shows the percentage viability of NIH-3T3 fibroblast cells after treatment with SHED-SM nanoemulgel. The results indicated that the highest cell viability was obtained in SHED H1 (SHED-SM H1) nanoemulgel at 65.95%, while the lowest was recorded for SHED-SM H5 at 37.01%. Statistical analysis using one-way ANOVA and LSD tests revealed significant differences in cell viability among SHED-SM H1, H3, and H5 (p < 0.05). Based on these findings, SHED-SM H1 nanoemulgel was selected for application in experimental animal studies.

**Figure 3.**
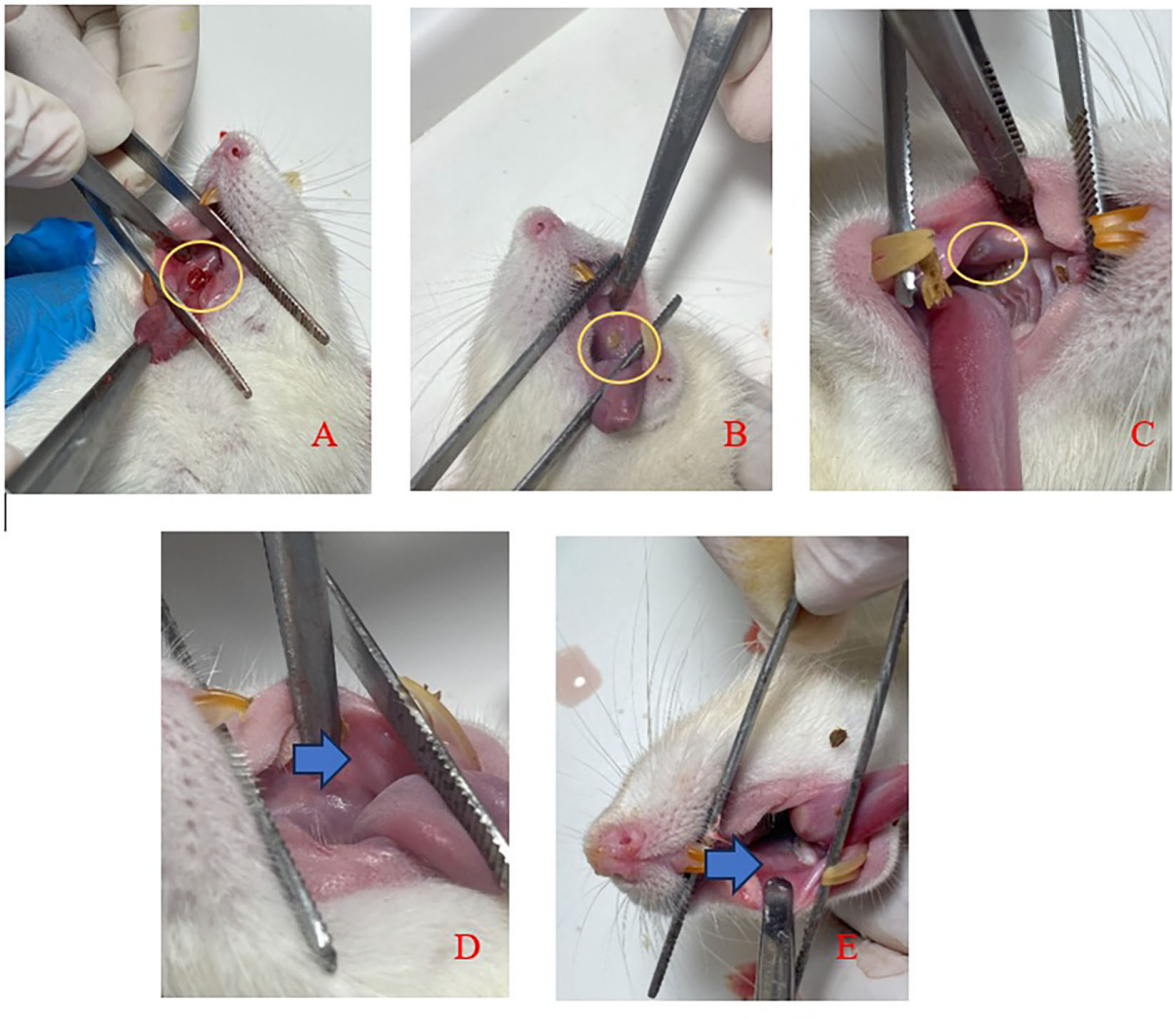
Clinical evaluation of traumatic ulcer after application of nanoemulgel spent medium SHED (SHED-SM) on the buccal mucosa of rats. An ulcer with a diameter of Ø 3 mm was seen on day 1 (A) and the diameter of the ulcer decreased as the observation days 3 ^rd^ (B) and 5 ^th^ (C). The ulcer had closed and could not be observed clinically on days 7 ^th^ (D) and 14 ^th^ (E).

Figure 3 displays clinical observations of the buccal mucosa following the application of SHED-SM nanoemulgel. By the 3 ^rd^ day, the traumatic ulcers in both the treatment and control groups showed closure, and a reduction in wound diameter over each observation day. On the 5 ^th^ day, ulcers were still visible but had decreased in size. By the 7 ^th^ and 14 ^th^ days, no ulcers were clinically observable.

**Figure 4.**
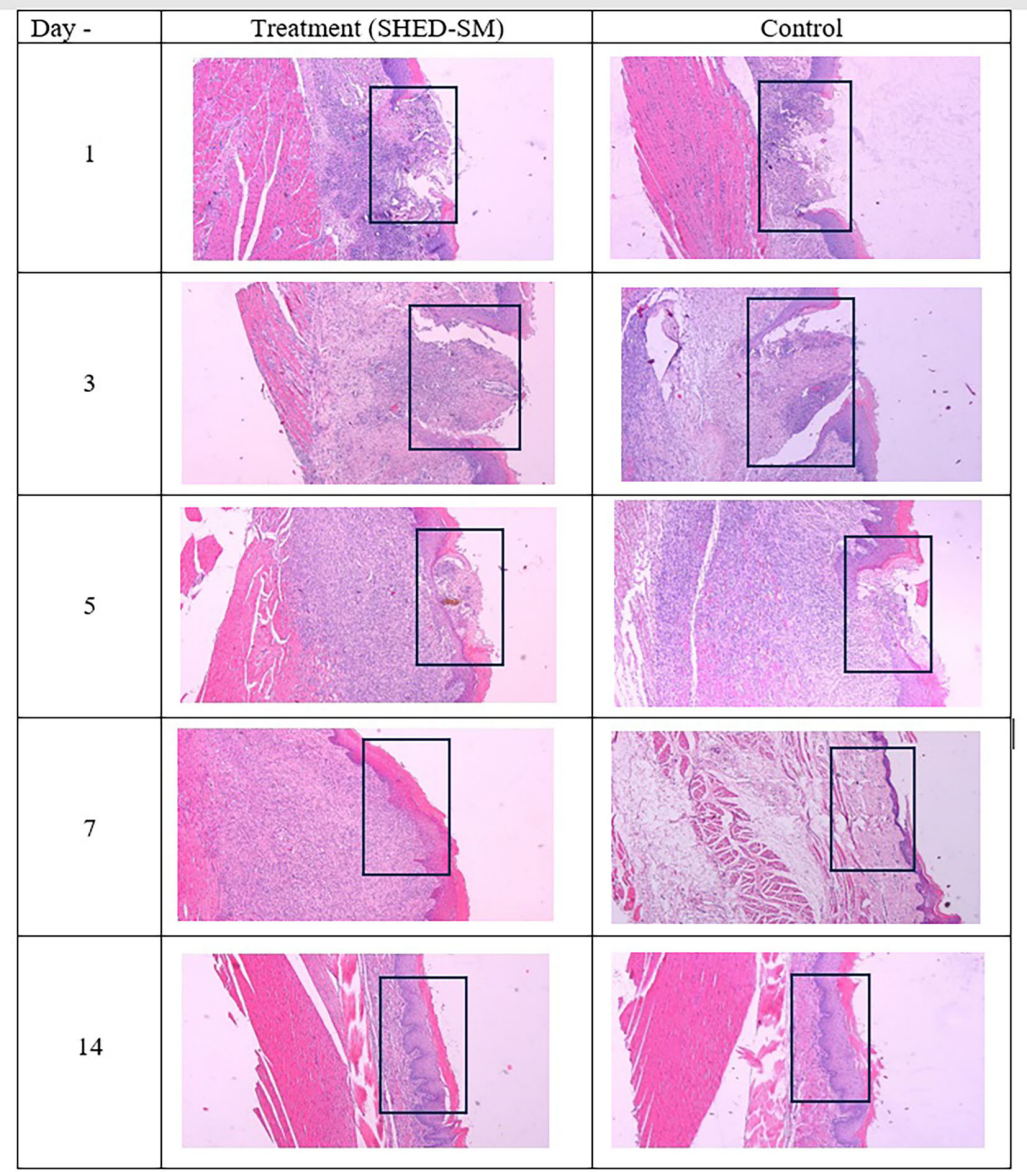
Evaluation of the wound healing process after application of nanoemulgel spent medium SHED (SHED-SM) (treatment) and control. Inflammatory cell infiltration appeared on 1 ^st^ and 3 ^rd^ day. The epithelium closed on the 5 ^th^ day after treatment, while the epithelium was still open in the control group. On the 7 ^th^ days and 14 ^th^, the epithelium has closed. Observation on the 7 ^th^ day of the treatment group showed thicker epithelium than the control group. Hematoxylin eosin staining at 40x magnification.

Figure 4 provides a histological evaluation of the healing process in traumatic ulcer wounds, comparing the treatment group (SHED-SM nanoemulgel) after application of spent medium SHED gel) and control (Aloclair Plus).

Figure 4 illustrates the ulcer healing process after applying treatment with SHED Spent Medium (SHED-SM) nanoemulgel compared to the control group treated with Aloclair Plus gel. On the 1 ^st^ day, inflammatory cell infiltration was seen until 3 ^rd^ day. On the 5 ^th^ day after treatment, epithelial closure was evident in the treatment group, while the control group demonstrated incomplete epithelial closure. On the 7 ^th^ and 14 ^th^ days, the ulcers were fully closed in both groups. On the 7 ^th^ day, the treatment group displayed thicker epithelial layers compared to the thinner epithelium in the control group. ^26^

**Figure 5.**
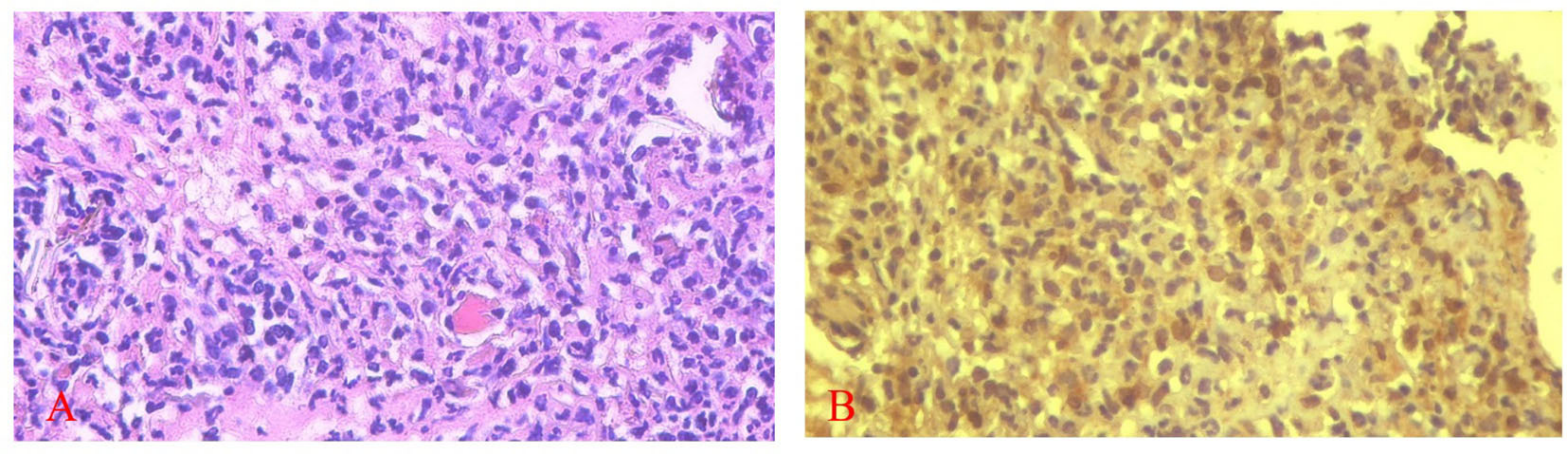
Neutrophil infiltration (A) and COX-2 expression (B) after application of nanoemulgel spent medium SHED (SHED-SM) on the 3 ^rd^ day. COX-2 expression was seen in the cytoplasm of cells. HE staining (A) and immunohistochemistry (B) at 400x magnification.

**Figure 6.**
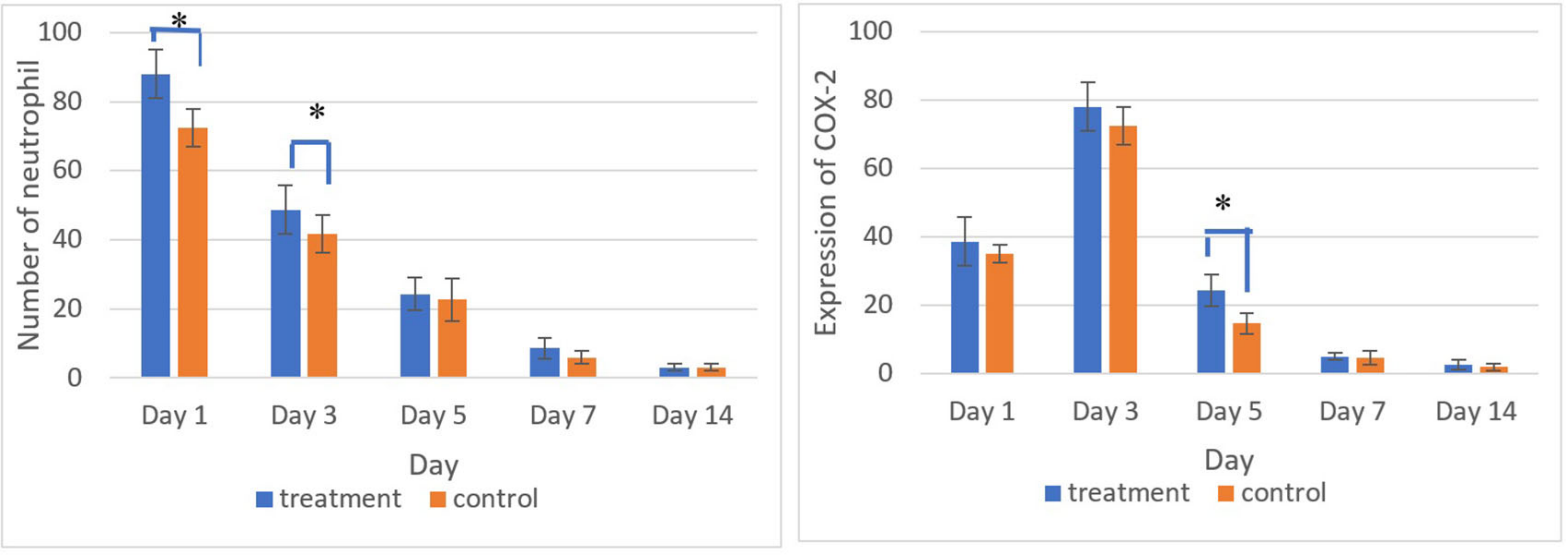
The number of neutrophils was highest on the 1 ^st^ day, while the positive expression of COX-2 on the 3 ^rd^ day decreased until the 14 ^th^. There was a significant difference in the number of neutrophils between the treatment and control group on the 1 ^st^ and 3 ^rd^ days and COX-2 expression on the 5 ^th^ day (*) (p<0.05).

Figures 5 and 6 present the results for neutrophil counts and COX-2 expression. The highest neutrophil count occurred on the 1 ^st^ day, while peak COX-2 expression was observed on the 3 ^rd^ day in both groups. Positive COX-2 expression was indicated by the presence of brown cytoplasmic staining in inflammatory cells within the ulcer area. Both neutrophil counts and COX-2 expression decreased progressively over the observation period.

The homogeneity and normality analyses of neutrophil count and COX-2 expression data confirmed normal and homogeneous distributions (p > 0.05). One-way ANOVA results indicated significant differences (p < 0.05), with LSD analysis revealing significant differences in neutrophil counts between the treatment and control groups on the 1 ^st^ and 3 ^rd^ days, as well as in COX-2 expression on the 5 ^th^ day.

**Figure 7.**
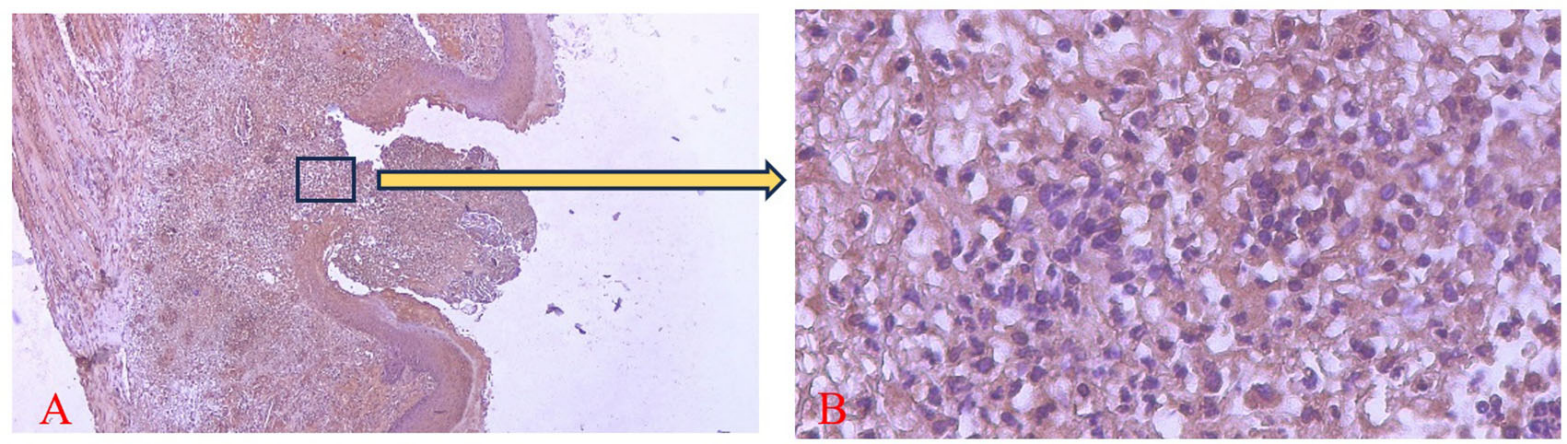
Evaluation of Collagen-1 (Col-1) expression on the 3 ^rd^ day after treatment showed a brown color in the extracellular matrix area and fibroblasts in the ulcer area. Magnification 40x (A) and 400x (B).

**Figure 8.**
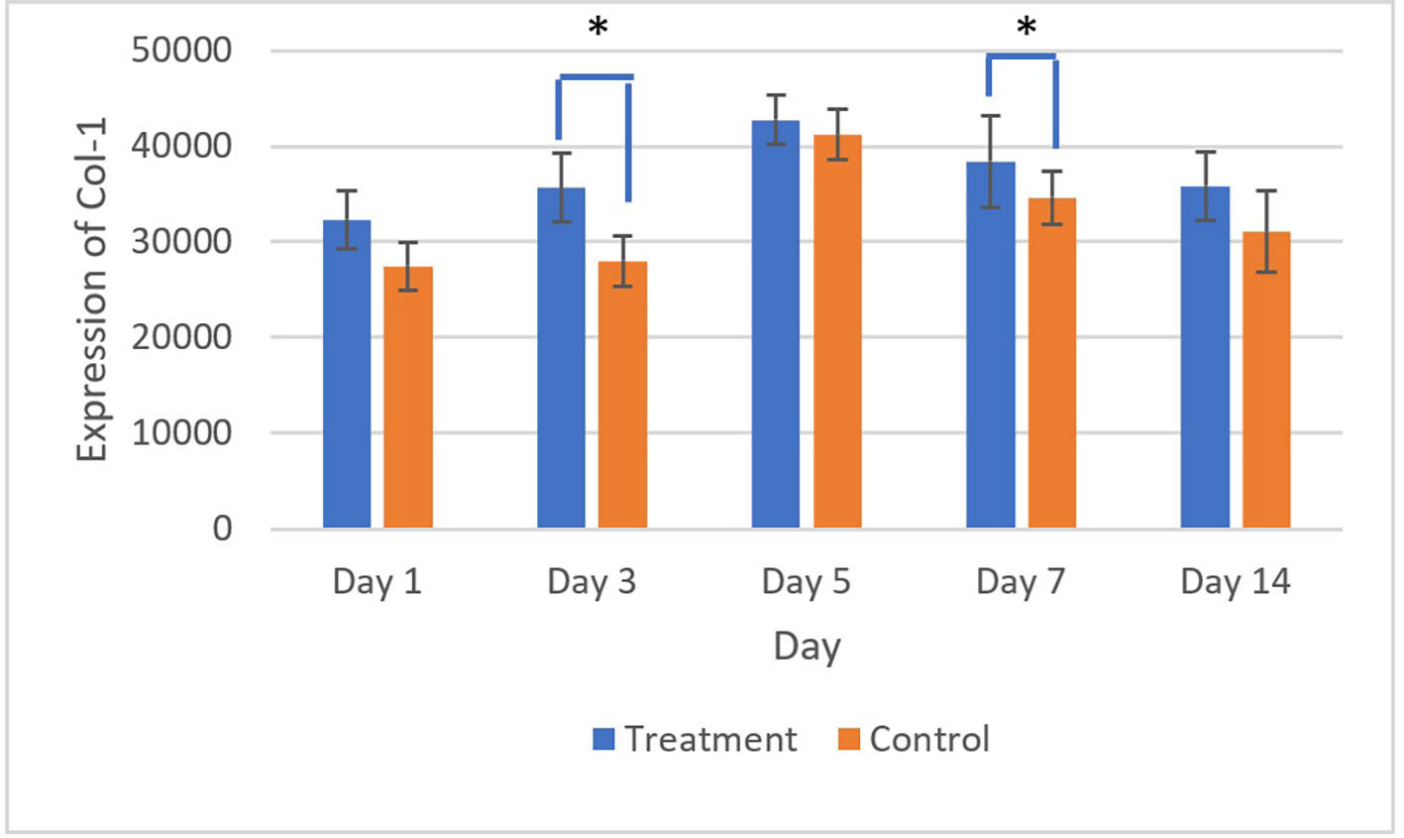
The highest Col-1 expression was shown on the 5 ^th^ day after treatment and control, and the expression decreased until the 14 ^th^ day. There was a significant difference in Col-1 expression on 3 ^rd^ and 5 ^th^ days (*) (p<0.05).

The brown staining observed in fibroblasts and the extracellular matrix, as shown in Figures 7 and 8, indicates collagen 1 (Col-1) expression. Homogeneity and normality tests for Col-1 expression data confirmed normal and homogeneous distributions (p > 0.05). One-way ANOVA results revealed significant differences (p < 0.05), and LSD analysis identified significant differences (p < 0.05) in Col-1 expression between the treatment and control groups on the 3 ^rd^ and 7 ^th^ days. ^26^

**Figure 9.**
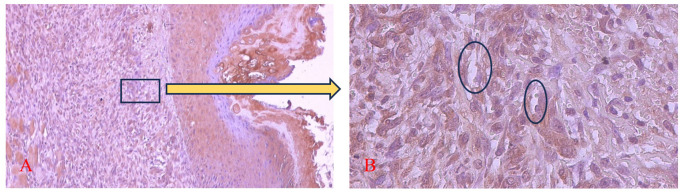
Evaluation of VEGF expression on the 5 ^th^ day after application of nanoemulgel spent medium SHED (SHED-SM). The brown color was seen in endothelial cells (Ο), fibroblasts, and inflammatory cells in the ulcer area. Magnification 100x (A) and 400x (B).

**Figure 10.**
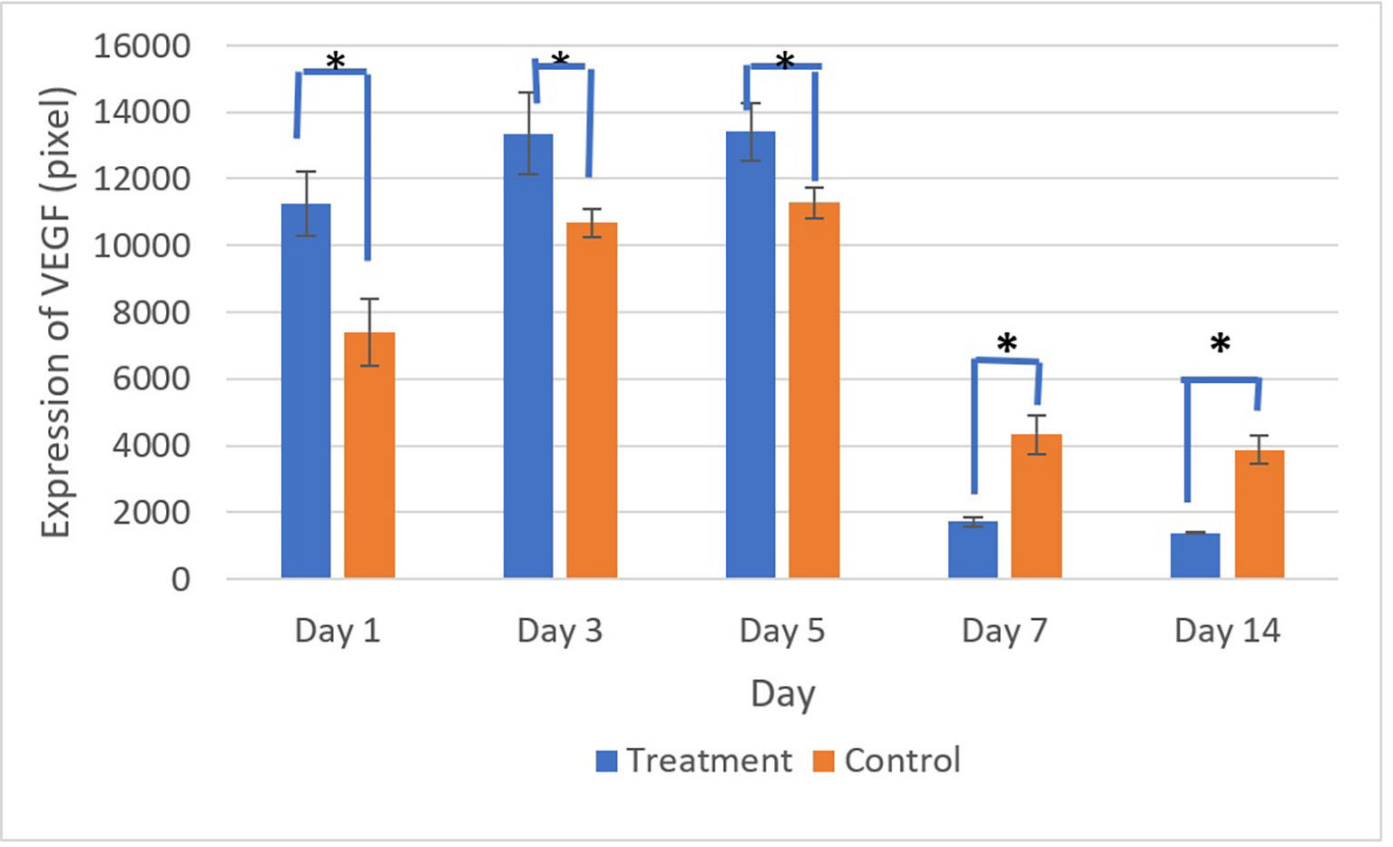
VEGF expression increased until the 5 ^th^ day in the control and treatment groups, then decreased until the 14 ^th^ day. There was a significant difference in VEGF expression between the control and treatment groups on all days (*) (p<0.05).

Figures 9 and 10 present the evaluation of VEGF expression in response to SHED-SM nanoemulgel and the control. VEGF expression, marked by brown staining in endothelial cells, fibroblasts, and inflammatory cells at the ulcer margins, was analyzed. ^25^

Homogeneity and normality tests confirmed normal and homogeneous data distributions (p > 0.05). One-way ANOVA results indicated significant differences (p < 0.05), with LSD analysis showing significant differences (p < 0.05) in VEGF expression between the treatment and control groups on all observation days.

This study demonstrates that SHED-SM nanoemulgel accelerates the healing of traumatic ulcers compared to the control. Clinically, this was evidenced by reduced ulcer size and epithelial closure by the 5 ^th^ day. Histological evaluation revealed that SHED-SM nanoemulgel treatment increased neutrophil counts on the 1 ^st^ day and COX-2 expression on the 3 ^rd^ day compared to the control. Additionally, Col-1 and VEGF expression levels were higher in the treatment group than in the control group, except on the 7 ^th^ and 14 ^th^ days, when VEGF expression was higher in the control group.

## Discussion

The viability cells test results revealed the highest cell viability ( Figures 1 and 2) obtained in SHED Spent Medium (SHED-SM) nanoemulgel H1, 65.95%. These results were used as the basis for application to experimental animals, specifically using SHED-SM nanoemulgel H1.

In the experimental animal treatment, clinical observations indicated that traumatic ulcers healed following both treatment and control applications. By the 5 ^th^ day, the ulcers were observed to have closed, and by the 7 ^th^ day, no ulcers were visible ( Figure 3). Histological observations using Hematoxylin Eosin staining corroborated these findings. In the treatment group, the epithelium appeared closed by the 5 ^th^ day, whereas in the control group, it remained open ( Figure 4).

Comparative analysis of neutrophil counts between the treatment and control groups showed the highest levels on the 1 ^st^ day. COX-2 expression peaked on the 3 ^rd^ day and decreased over subsequent observation days. COX-2 expression plays a critical role in the ulcer healing process, as it is known to increase rapidly within 12 hours of mucosal injury. The COX-2 enzyme is essential for producing prostaglandin E2 (PGE2), a key mediator of inflammation. The sharp increase in COX-2 expression on the 3 ^rd^ day indicates active debris and pathogen clearance in the ulcer area. By the 5 ^th^ day and in later observations, a decline in COX-2 expression was evident. This decline likely marks the transition to the proliferation phase of healing. Additionally, reduced COX-2 expression helps mitigate excessive inflammation and supports the formation of new tissue. ^19^

The observed decrease in the number of neutrophil cell infiltration and COX-2 expression in the SHED-SM treatment group and the Aloclair Plus ^®^ control group was likely due to the anti-inflammatory effects of both components. The anti-inflammatory effects of SHED-SM may stem from its bioactive content, which could modulate the inflammatory response, including reducing the production of pro-inflammatory cytokines (TNF-α and IL-6). ^19^ However, further research is still needed to prove this mechanism. Similarly, in the control group, the reduction in the number of neutrophils and COX-2 expression could be attributed to the anti-inflammatory effects of Aloclair Plus ^®^ ingredients, including Aloe Vera, Sodium Hyaluronate, Glycyrrhetinic Acid, and Polyvinylpyrrolidone (PVP). The four ingredients were likely to reduce inflammation, especially PVP, which can form a layer on the wound’s surface, thereby preventing further ulcer irritation.

The evaluation of Collagen-1 (Col-1) expression showed the highest expression on the 5 ^th^ day after treatment and control, followed by a gradual decline until the 14 ^th^ day. Collagen-1 was known to have an important role in the mucosal wound healing process. The increase in Col-1 expression was in line with the new tissue formation and remodeling process. Figure 8 showed a rise in Col-1 expression from the 1 ^st^ to 5 ^th^ days. This result was thought to be in the early stages of ulcer healing (inflammatory phase on 1 ^st^ and 3 ^rd^ days); col-1 played a role in strengthening the extracellular matrix and supporting the migration of inflammatory cells to the ulcer area. On the 5 ^th^ day, Col-1 expression continued to increased, likely marking the proliferation phase, during which fibroblasts migrate to the ulcer area and synthesize collagen, forming new granulation tissue. This day also marks the peak of Col-1 expression. Col-1 expression decreased in the remodeling phase, suggesting that the tissue had matured and become more organized.

Evaluation of VEGF expression revealed increased levels on the 3 ^rd^ and 5 ^th^ days, with higher expression in the treatment group compared to the control. Conversely, on the 7 ^th^ and 14 ^th^ days, VEGF expression in the control group was higher than in the treatment group. These results indicated that VEGF expression plays an important role in the proliferation phase by stimulating the growth of new blood vessels to replace ulcer damage. Strong VEGF expression was seen in endothelial cells, fibroblasts, and inflammatory cells. VEGF expression in endothelial cells contributing to cell proliferation and migration to form new capillaries.

The SHED-SM material can be applied topically to oral mucosal ulcer wounds. The advantages of the topical method may have high bioavailability and is more selective and efficient in drug delivery. ^20^ One factor affecting wound healing therapy was the particle size of the material used. Small particles were easier to absorb into the body. However, the disadvantage of small particles is that they cannot last long in wounds. ^21^ The development of nanoemulsion-based formulations was known to increase the absorption of therapeutic materials into the skin. This particle shape can also carry hydrophilic and hydrophobic therapeutic materials. Gel preparations have a faster dissolution ability than cream or ointment preparations. ^22^ However, gels also have limitations, such as potential irritation from additional gel-forming agents, a high susceptibility to microbial or fungal contamination, and drying due to solvent evaporation. ^23^ These challenges can be mitigated by converting the formulation into a nanoemulgel. Nanoemulgels combine the benefits of nanoemulsion and gel, offering improved therapeutic agent release characteristics and a stable gel matrix consistency. As a result, nanoemulgel is considered an effective dosage form for delivering therapeutic agents through the skin. ^24, 25^

The results of this study indicated that the application of SHED-SM nanoemulgel can accelerate the healing of traumatic ulcers. This was characterized by a smaller ulcer diameter on the 5 ^th^ day, supported by the results of histological evaluation in the form of epithelial closure, the number of neutrophils on the 1 ^st^ day followed by COX-2 expression on the 3 ^rd^ day, expression of Col-1 and VEGF. All the parameters were consistently higher in the treatment group compared to the control.

The mechanism of SHED-SM in accelerating the healing of traumatic mucosal ulcers includes: 1) SHED-SM may deliver a concentrated mixture of soluble growth factors, cytokines, chemokines, extracellular vesicles (including exosomes), and regulatory RNAs that act paracrinally to orchestrate each phase of wound healing, 2) Components of SHED-SM may recruit neutrophils and monocytes (marked by COX-2 expression), secreted antimicrobial peptides and protease inhibitors reduce bacterial burden and preserve provisional matrix integrity, 3) SHED-SM bioactive cytokines (including TGF β) and exosomal miRNAs could enhance macrophage polarization from M1 to M2, decrease TNF α and IL 1β levels and increase pro-repair mediators, 4) SHED-SM growth factors (VEGF, FGF) and exosome may stimulate endothelial cell proliferation and sprouting, fibroblast proliferation and migration, keratinocyte re-epithelialization, and myofibroblast differentiation, 5) The TGF β content in SHED-SM may modulate collagen synthesis (as indicated by increased Col-1 expression) and MMP-mediated remodeling to increase tensile strength and reduce fibrosis.

In conclusion, the application of SHED Spent Medium nanoemulgel could accelerate the healing of traumatic buccal mucosal ulcers, as demonstrated by clinical and histological outcomes, including epithelial closure and increased neutrophil count, as well as elevated expressions of COX-2, Col-1, and VEGF.

## Data Availability

Handajani, Juni (2025). Raw Data RKI-2024 https://figshare.com/articles/dataset/Raw_Data_RKI-2024/28816256. ^26^ https://doi.org/10.6084/m9.figshare.28816256.v1. ^26^ Data are available under the terms of the Creative Commons Attribution 4.0 International license (CC-BY 4.0).
