# Supplementary material for: Effect of Stem Cells from Human Exfoliated Deciduous Teeth Spent Medium (SHED-SM) on the Healing of Traumatic Buccal Mucosal Ulcers
Source: F1000Res. 2025 Oct 21;14:510. Originally published 2025 May 21. [Version 2] doi: 10.12688/f1000research.164339.2 (PMC12514440; doi:10.12688/f1000research.164339.2)
Supplement: Peer review history

# Reviewer report: Reviewer response for version 2

**DOI:** 10.5256/f1000research.189895.r425811

**Published:** 2025-10-31

**Authors:** Dhona Afriza

**Affiliations:** 1 Baiturrahmah University, Sumatera Barat, Indonesia

The manuscript has undergone a comprehensive review. The authors have effectively responded to all prior comments and made the recommended revisions accordingly. The manuscript has significantly enhanced in clarity and quality. I have no additional comments and deem the manuscript appropriate for indexing as it currently stands.

Is the work clearly and accurately presented and does it cite the current literature?

Yes

If applicable, is the statistical analysis and its interpretation appropriate?

I cannot comment. A qualified statistician is required.

Are all the source data underlying the results available to ensure full reproducibility?

Yes

Is the study design appropriate and is the work technically sound?

Yes

Are the conclusions drawn adequately supported by the results?

Yes

Are sufficient details of methods and analysis provided to allow replication by others?

Yes

Reviewer Expertise:

Biology oral, molecular biology, molecular dynamics, oral cancer

I confirm that I have read this submission and believe that I have an appropriate level of expertise to confirm that it is of an acceptable scientific standard.

# Reviewer report: Reviewer response for version 1

**DOI:** 10.5256/f1000research.180835.r419331

**Published:** 2025-10-13

**Authors:** Euis Reni Yuslianti

**Affiliations:** 1 Universitas Jenderal Achmad Yani, Cimahi, West Java, Indonesia

-The study design is good, but the abstract and the methods should clearly mention the type of experimental design used, explain how the animals were divided or randomized, and clarify whether the cell and animal tests were done separately or as part of one study to make the methods easier to understand and repeat.

 -The introduction provides good background information, but it should be made more concise, avoid repetition about stem cell functions, and clearly emphasize the research gap and novelty of using SHED-SM nanoemulgel for buccal mucosal ulcer healing.

 -The discussion is informative and well-structured, but as a reviewer, I would suggest:

 -The discussion effectively interprets the findings; however, it could be strengthened by linking the molecular mechanisms more directly to the study’s results, minimizing descriptive repetition of known pathways (e.g., COX-2, VEGF), and providing a clearer comparison with previous studies to highlight the novelty and clinical relevance of SHED-SM nanoemulgel in mucosal ulcer healing.

Is the work clearly and accurately presented and does it cite the current literature?

Yes

If applicable, is the statistical analysis and its interpretation appropriate?

Yes

Are all the source data underlying the results available to ensure full reproducibility?

Yes

Is the study design appropriate and is the work technically sound?

Yes

Are the conclusions drawn adequately supported by the results?

Yes

Are sufficient details of methods and analysis provided to allow replication by others?

Yes

Reviewer Expertise:

Biochemical and biomolecullar

I confirm that I have read this submission and believe that I have an appropriate level of expertise to confirm that it is of an acceptable scientific standard.

# Response

**Published:** 2025-10-17

**Authors:** Juni Handajani

**Affiliations:** Universitas Gadjah Mada, Indonesia

Thank you for your insightful suggestion; the manuscript has been revised to incorporate the requested clarifications.

# Reviewer report: Reviewer response for version 1

**DOI:** 10.5256/f1000research.180835.r419329

**Published:** 2025-10-10

**Authors:** Zahreni Hamzah

**Affiliations:** 1 University of Jember, Jember, Indonesia

**Tittle. **This title is already quite solid, clear, interesting, and relevant in the context of current research in the field of stem cells.

**Abstract. **The abstract you have written is quite good, clear, and concise.

**Introduction**

**1. **Regenerative therapies using stem cells are currently being developed to accelerate wound healing (written twice, please remove one instance).

**2. **SHED-SM nanoemulgel (at the end of the introduction) **, **please clarify the nanoemulgel in relation to secretome therapy.

**3. **Provide a brief explanation of the mechanism of action of secretomes in the wound healing process, with more specific details on wound healing.

**Material and Methods**

 This section is described in great detail and methodologically, providing a clear description of the experimental design and protocols used, including cell isolation, cytotoxicity testing, animal experiments, and histological analysis. However, additional explanation is needed regarding the randomization of animal grouping.

**Results**

 This study presents clear results, relevant images, accurate statistical analysis, and in-depth evaluation of the network, as well as measurement of relevant parameters, indicating that SHED-SM nanoemulgel can accelerate the healing of traumatic ulcers more effectively. This study has the potential to make a significant contribution to the development of regenerative therapies for wound healing. However, further discussion is needed on the role of VEGF and COX-2 in the wound healing process.

Is the work clearly and accurately presented and does it cite the current literature?

Yes

If applicable, is the statistical analysis and its interpretation appropriate?

Yes

Are all the source data underlying the results available to ensure full reproducibility?

Yes

Is the study design appropriate and is the work technically sound?

Yes

Are the conclusions drawn adequately supported by the results?

Yes

Are sufficient details of methods and analysis provided to allow replication by others?

Yes

Reviewer Expertise:

Oral Biology, Physiology, Physio-Geriatry Oromaxillofacial, Stress

I confirm that I have read this submission and believe that I have an appropriate level of expertise to confirm that it is of an acceptable scientific standard.

# Response

**Published:** 2025-10-17

**Authors:** Juni Handajani

**Affiliations:** Universitas Gadjah Mada, Indonesia

We appreciate the constructive comment and have modified the manuscript accordingly to resolve the points mentioned.

# Reviewer report: Reviewer response for version 1

**DOI:** 10.5256/f1000research.180835.r415719

**Published:** 2025-10-08

**Authors:** Dhona Afriza

**Affiliations:** 1 Baiturrahmah University, Sumatera Barat, Indonesia

The study is relevant and well designed; however, some clarifications are needed. The statistical analysis is only described as “ANOVA,” without specifying the exact type used, post hoc procedures, or assumption tests. These details should be reported in the Methods section, while the Results should present outcomes only. In addition, the MTT assay is presented as a cytotoxicity test, but the data reflect cell viability; given the focus on fibroblast survival, it would be clearer to describe this as a cell viability test. Clarifying these points will improve transparency and strengthen the validity of the conclusions.

Is the work clearly and accurately presented and does it cite the current literature?

Yes

If applicable, is the statistical analysis and its interpretation appropriate?

I cannot comment. A qualified statistician is required.

Are all the source data underlying the results available to ensure full reproducibility?

Yes

Is the study design appropriate and is the work technically sound?

Yes

Are the conclusions drawn adequately supported by the results?

Yes

Are sufficient details of methods and analysis provided to allow replication by others?

Yes

Reviewer Expertise:

Biology oral, molecular biology, molecular dynamics, oral cancer

I confirm that I have read this submission and believe that I have an appropriate level of expertise to confirm that it is of an acceptable scientific standard.

# Response

**Published:** 2025-10-17

**Authors:** Juni Handajani

**Affiliations:** Universitas Gadjah Mada, Indonesia

Thank you for the constructive comment. We have revised the manuscript to address the points raised.
